# Integrated ATAC-Seq and RNA-Seq Reveal Candidate Regulatory Genes and Chromatin Accessibility Associated with Intramuscular Fat Deposition: An Animal Trial in Hezuo Pigs

**DOI:** 10.3390/ani16142172

**Published:** 2026-07-13

**Authors:** Jiaojiao Yang, Xiaoyu Huang, Qiaoli Yang, Jie Li, Shuangbao Gun

**Affiliations:** 1 College of Animal Science and Technology, Gansu Agricultural University, Lanzhou 730070, China; yangjj@gsau.edu.cn (J.Y.); huangxy@gsau.edu.cn (X.H.); yangql@gsau.edu.cn (Q.Y.); lijie5272@126.com (J.L.); 2 Gansu Province Modern Pig Engineering Technology Innovation Center, Lanzhou 730070, China

**Keywords:** Hezuo pig, intramuscular fat, longissimus dorsi muscle, multi-omics integration, chromatin accessibility, lipid metabolism, indigenous breed

## Abstract

Intramuscular fat content is a key determinant of pork quality, influencing traits such as tenderness, juiciness, and flavor. However, the molecular mechanisms regulating intramuscular fat deposition in indigenous pig breeds remain incompletely understood. This study aimed to identify genes and regulatory mechanisms associated with intramuscular fat accumulation in Hezuo pigs. Longissimus dorsi muscle samples from Hezuo pigs with extreme high and low intramuscular fat contents were subjected to chromatin accessibility profiling and transcriptome sequencing. Comparative analyses identified 2201 differentially accessible chromatin regions and 588 differentially expressed genes between the two groups. Functional enrichment analyses indicated that these genes were mainly involved in lipid metabolism, focal adhesion, extracellular matrix–receptor interaction, fatty acid metabolism, and adenosine monophosphate-activated protein kinase signaling. Integration of chromatin accessibility and gene expression datasets identified 92 co-regulated genes associated with intramuscular fat deposition, including *MYLK3*, *PDGFC*, *PAK1*, *IGF1R*, *LAMA4*, *DIAPH1*, *SDC4*, *GADD45G*, and *RXRG*. These findings reveal regulatory networks underlying intramuscular fat accumulation in Hezuo pigs and provide candidate genes and molecular resources for improving meat quality through genetic selection and breeding programs.

## Simple Summary

Intramuscular fat is an important factor affecting pork quality because it contributes to meat tenderness, juiciness, flavor, and marbling. However, the biological processes that control fat deposition within muscle are not fully understood, particularly in indigenous pig breeds with desirable meat quality traits. In this study, we investigated the longissimus dorsi muscle of Hezuo pigs with naturally high or low levels of intramuscular fat. By combining two genome-wide approaches that examine gene activity and DNA regulatory regions, we identified genes and biological pathways associated with differences in fat accumulation. Several genes involved in energy metabolism, cell signaling, and tissue development were found to be closely related to intramuscular fat deposition. We also identified regulatory factors that may influence how these genes are activated. These findings improve our understanding of the genetic mechanisms underlying fat deposition in pig muscle and provide valuable information for the development of breeding strategies aimed at improving pork quality. Ultimately, this research helps producers breed pigs with superior meat quality, provides consumers with better pork products, and offers valuable insights for human biomedical research into metabolic diseases.

## 1. Introduction

Pigs are one of the most important livestock species and represent a major source of animal protein for human consumption. With continuous improvements in living standards, consumer demand for high-quality pork has increased substantially. Meat quality is a complex trait influenced by multiple factors, including intramuscular fat (IMF) content, pH, tenderness, and meat color [1,2]. Among these factors, IMF content plays a critical role in determining pork quality by affecting flavor, tenderness, juiciness, and overall eating quality [3,4,5,6,7]. In addition to its importance in animal production, IMF deposition has attracted considerable attention in biomedical research. Because pigs share many anatomical and physiological characteristics with humans, they serve as valuable animal models for studying human diseases [8,9]. Moreover, abnormal fat deposition is closely associated with metabolic disorders in humans. Therefore, identifying the genes that regulate IMF deposition and elucidating the underlying molecular mechanisms are important not only for improving pork quality through genetic breeding but also for advancing our understanding of fat metabolism and related human diseases.

Advances in high-throughput sequencing and multi-omics technologies have greatly facilitated the identification of genetic variants and candidate genes associated with complex economic traits in livestock through transcriptomic, genomic, and epigenomic analyses [10,11]. IMF deposition is a complex polygenic trait regulated by multiple genetic and environmental factors. Recent studies have identified numerous genes and regulatory molecules associated with IMF variation in pigs. For example, Wang et al. demonstrated that MED17 acts as a novel regulator of IMF content through integrated genomic and transcriptomic analyses [12]. Tang et al. identified key genes and long non-coding RNAs involved in IMF deposition using weighted gene co-expression network analysis, functional enrichment analysis, and protein–protein interaction network analysis [13]. Likewise, Zhang et al. revealed candidate genes associated with IMF deposition through multi-omics integration of longissimus dorsi muscle tissues from different pig breeds [14]. Although several genes related to porcine IMF deposition, including *PPARG*, *PLIN1*, and *FABP4*, have been reported [15], most previous studies have primarily focused on transcriptomic, proteomic, or metabolomic analyses. In contrast, integrative studies combining chromatin accessibility and gene expression profiling remain limited, particularly in indigenous pig breeds. As a result, the key regulatory genes and transcription factors underlying IMF deposition have not been fully elucidated, which restricts the application of molecular markers in pig breeding programs aimed at improving meat quality.

Hezuo pigs are a distinctive indigenous Chinese breed known for their superior meat quality and strong environmental adaptability. However, the molecular mechanisms underlying IMF deposition in this breed remain poorly understood. Therefore, this study aimed to identify key genes associated with IMF deposition and to elucidate the underlying regulatory mechanisms. To achieve this objective, IMF content was quantified in the longissimus dorsi muscle of Hezuo pigs, and individuals with extreme IMF phenotypes were selected for chromatin accessibility and transcriptome profiling. ATAC-seq and RNA-seq analyses were subsequently performed to identify differentially accessible chromatin regions (DACRs) and differentially expressed genes (DEGs) associated with IMF variation. By integrating these datasets, we identified candidate regulatory genes and pathways potentially involved in IMF deposition. The findings of this study provide valuable insights into the genetic and epigenetic mechanisms governing IMF accumulation in pigs and offer molecular resources for future breeding strategies aimed at improving pork quality.

## 2. Materials and Methods

### 2.1. Tissue Sampling and IMF Determination

Sixty healthy Hezuo pigs at 90 days of age were selected from the same commercial farm and raised under identical feeding and management conditions. All animals were slaughtered at 90 days of age, and longissimus dorsi muscle samples were collected immediately after slaughter. Each sample was divided into two portions: approximately 200 g was stored at −20 °C for IMF determination, whereas approximately 5 g was snap-frozen in liquid nitrogen and stored at −80 °C for subsequent molecular analyses.

IMF content was measured using a FoodScan Meat Analyzer (FOSS Analytical A/S, Hillerød, Denmark) according to the manufacturer’s instructions. Briefly, longissimus dorsi muscle samples were minced and thoroughly homogenized, and the homogenates were loaded into the sample tray for analysis. Based on the IMF measurements, six male pigs with similar body weights were selected for further study and assigned to either a high-IMF group (*n* = 3) or a low-IMF group (*n* = 3). These samples were subsequently subjected to ATAC-seq and RNA-seq analyses.

### 2.2. RNA Extraction, Library Preparation, and Sequencing

Total RNA was extracted from longissimus dorsi muscle samples collected from Hezuo pigs with high and low IMF contents using TRIzol Reagent (Cat. No. 15596026; Invitrogen, Carlsbad, CA, USA) following the manufacturer’s protocols. RNA concentration, purity, and integrity were assessed using an Agilent 2100 Bioanalyzer (Agilent Technologies, Santa Clara, CA, USA). High-quality RNA samples were enriched using poly-T oligo-attached magnetic beads to isolate messenger RNA. First-strand cDNA was synthesized using random hexamer primers, followed by second-strand cDNA synthesis. Sequencing libraries were subsequently constructed through end repair, A-tailing, adapter ligation, fragment size selection, PCR amplification, and purification. Library quality and concentration were evaluated using a Qubit Fluorometer (Thermo Fisher Scientific, Waltham, MA, USA) and real-time PCR, whereas fragment size distribution was assessed using the Agilent 2100 Bioanalyzer (Agilent Technologies). The qualified libraries were sequenced on an Illumina NovaSeq 6000 platform (Illumina, San Diego, CA, USA) by Tianjin Novogene Bioinformatics Technology Co., Ltd. (Tianjin, China).

### 2.3. RNA-Seq Data Preprocessing

Raw FASTQ reads were processed using fastp (v0.20.0) with default parameters to remove adapter-contaminated reads, poly-N-containing reads, and low-quality sequences. The quality of the clean data was assessed by calculating Q20, Q30, and GC content values. Clean reads were then aligned to the pig reference genome (Sus scrofa 11.1; https://www.ncbi.nlm.nih.gov/datasets/genome/GCF_000003025.6/, accessed on 16 March 2025) using HISAT2 (v2.0.5). Gene-level read counts were generated using featureCounts (v1.5.0-p3), and gene expression levels were normalized and quantified as fragments per kilobase of transcript per million mapped reads (FPKM) for subsequent analyses.

### 2.4. Identification and Functional Enrichment Analysis of DEGs

Differential expression analysis between the high- and low-IMF groups was performed using the DESeq2 package (v1.20.0) in R (v4.3.3). DEGs were identified using thresholds of *q*-value < 0.05 and |log_2_(fold change)| ≥ 1. Functional enrichment analysis of DEGs was conducted based on the Kyoto Encyclopedia of Genes and Genomes (KEGG) database using the clusterProfiler package (v3.8.1) in R. Pathways with *p* < 0.05 were regarded significantly enriched.

### 2.5. Validation of DEGs

Quantitative real-time PCR (qRT-PCR) was performed to validate the expression patterns of DEGs identified by RNA-seq. Six DEGs were randomly selected, and gene-specific primers were designed using the NCBI Primer-BLAST online tool. Primer sequences are provided in Supplementary Table S1. Total RNA was extracted from longissimus dorsi muscle samples obtained from three high-IMF and three low-IMF pigs using TRIzol Reagent (Cat. No. 15596026; Invitrogen). First-strand cDNA was synthesized using the HiScript IV All-in-One Ultra RT SuperMix for qPCR (Cat. No. R333-01; Vazyme Biotech Co., Ltd., Nanjing, China) according to the manufacturer’s protocols. qRT-PCR was conducted using SupRealQ Ultra Hunter SYBR qPCR Master Mix (U+) (Cat. No. Q142-02; Vazyme Biotech Co., Ltd., Nanjing, China) on a CFX384 Real-Time PCR Detection System (Bio-Rad Laboratories, Hercules, CA, USA). Each sample was analyzed with three technical replicates. Relative gene expression levels were normalized to *GAPDH* and calculated using the 2^−ΔΔCt^ method [16].

### 2.6. ATAC-Seq Library Construction and Sequencing

ATAC-seq libraries were prepared with minor modifications according to a previously described protocol [17]. Briefly, approximately 5 mg of frozen longissimus dorsi muscle tissue was ground in liquid nitrogen and resuspended in 1 mL of pre-chilled phosphate-buffered saline (PBS) to generate a cell suspension. Nuclei were isolated from the suspension and incubated with Tn5 transposase at 37 °C for 30 min to perform chromatin tagmentation. Following transposition, equal volumes of Adapter 1 and Adapter 2 were added, and the libraries were amplified by PCR using the Novogene ATAC-Seq Library Preparation Kit (Novogene). PCR products were purified using AMPure XP Beads (Beckman Coulter, Brea, CA, USA), and library quality and concentration were assessed using a Qubit Fluorometer (Thermo Fisher Scientific). Qualified libraries were sequenced on an Illumina NovaSeq 6000 platform (Illumina) by Tianjin Novogene Bioinformatics Technology Co., Ltd., generating 150-bp paired-end reads for subsequent analyses.

### 2.7. ATAC-Seq Data Analysis

ATAC-seq data were analyzed according to the ENCODE Project guidelines (https://www.encodeproject.org/, accessed on 20 March 2025) with minor modifications. Raw FASTQ reads were processed using fastp (v0.20.0) to remove adapter-contaminated sequences, low-quality reads, and poly-N-containing reads. The quality of the clean data was evaluated by calculating Q20, Q30, and GC content values. Clean reads were aligned to the pig reference genome (Sus scrofa 11.1; GCF_000003025.6) using BWA-MEM (v0.7.12-r1039). To improve mapping reliability, mitochondrial reads, improperly paired reads, and PCR duplicates were removed. Only uniquely mapped reads with a mapping quality (MAPQ) score ≥ 13 were retained for downstream analyses.

Accessible chromatin peaks were identified for each sample using MACS2 (v2.1.0) with the following parameters: -g 2.6 × 10^9^ -q 0.05 --call-summits --nomodel --shift -100 --extsize 200 --keep-dup all. Peak annotation was performed using the ChIPseeker (v1.38.0) package in R. To identify DACRs, peaks from all samples were merged using BEDTools (v2.29.2), and the average reads per million mapped reads (RPM) values of merged peaks were calculated for each group. Differential peaks were identified using thresholds of |log_2_(fold change)| ≥ 1 and *p* < 0.05. Genes associated with DACRs were subsequently annotated using ChIPseeker (v1.38.0).

### 2.8. Integrated Analysis of ATAC-Seq and RNA-Seq Datasets

ATAC-seq and RNA-seq datasets were integrated to investigate the relationship between chromatin accessibility and gene expression associated with IMF deposition. Briefly, genes associated with accessible chromatin regions identified by ATAC-seq were compared with the DEGs identified by RNA-seq. Upregulated and downregulated DEGs were subsequently overlapped with genes associated with gained and lost DACRs, respectively. Venn diagrams were generated to identify genes exhibiting concordant changes in chromatin accessibility and transcriptional activity. Co-regulated genes showing consistent differential patterns across the two datasets were subjected to KEGG pathway enrichment analysis. Significantly enriched pathways were identified using a threshold of *p* < 0.05. The enrichment results were visualized using Sankey dot plots generated with the SRplot online platform (https://www.bioinformatics.com.cn/; accessed on 4 May 2026) [18]. In addition, the Integrative Genomics Viewer (IGV; Broad Institute, Cambridge, MA, USA) was used to visualize chromatin accessibility signals at representative candidate gene loci.

### 2.9. Statistical Analysis

Phenotypic data are presented as mean ± standard deviation (SD). Differences in IMF content and body weight between the high- and low-IMF groups were evaluated using two-tailed Student’s *t*-tests. Statistical significance was defined as *p* < 0.05.

## 3. Results

### 3.1. Phenotypic Characteristics of Hezuo Pigs with Divergent IMF Content

Based on IMF measurements, six Hezuo pigs exhibiting divergent IMF phenotypes were selected for subsequent omics analyses, including three animals with high IMF content and three with low IMF content (Supplementary Table S2). The phenotypic characteristics of the two groups are summarized in Table 1. The mean IMF content of the high-IMF group was 3.64 ± 0.18%, which was significantly greater than that of the low-IMF group (2.03 ± 0.11%; *p* = 0.0004). In contrast, no significant difference in body weight was observed between the two groups (15.53 ± 2.05 kg vs. 13.83 ± 1.35 kg; *p* = 0.38). These results confirmed the successful selection of animals with markedly different IMF contents while minimizing potential confounding effects of body weight.

### 3.2. Quality Assessment of ATAC-Seq Data

To characterize chromatin accessibility associated with IMF variation, ATAC-seq was performed on longissimus dorsi muscle samples from Hezuo pigs with divergent IMF phenotypes. A total of 255,512,398 and 280,639,027 raw reads were generated from the high- and low-IMF groups, respectively. After adapter trimming and quality filtering, 252,106,860 clean reads were retained for the high-IMF group and 276,991,910 for the low-IMF group. Across all samples, the average Q20 and Q30 values reached 98.23% and 94.82%, respectively. Clean reads were subsequently aligned to the porcine reference genome, yielding non-mitochondrial mapping rates ranging from 93.19% to 95.80% and unique mapping rates ranging from 89.99% to 92.05% (Table 2).

**Figure 1:**
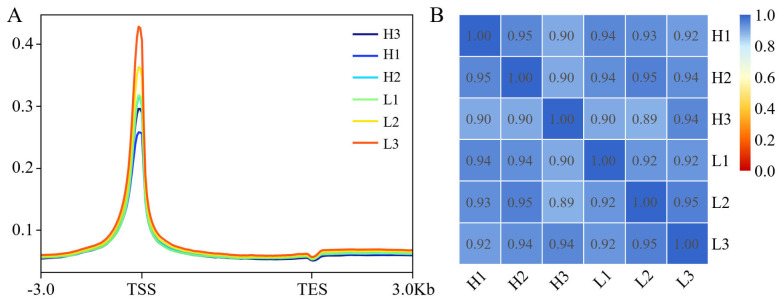
Quality control of ATAC-seq data. (**A**) Enrichment of ATAC-seq signals around transcription start sites. (**B**) Pearson correlation coefficients among biological replicates.

Library quality was further assessed by examining insert size distributions and chromatin accessibility signal profiles. All libraries exhibited the characteristic periodic pattern of open chromatin, with clear enrichment of nucleosome-free and mononucleosomal fragments (Figure S1). Moreover, accessibility signals were strongly enriched around TSSs (Figure 1A), consistent with the expected distribution of regulatory elements. Pearson correlation coefficients among biological replicates ranged from 0.92 to 0.96 (Figure 1B), indicating high reproducibility between samples. These results demonstrate the high quality and reliability of the ATAC-seq data for subsequent analyses of chromatin accessibility.

### 3.3. Genomic Characteristics of ACRs in the Longissimus Dorsi Muscle of Hezuo Pigs

**Figure 2:**
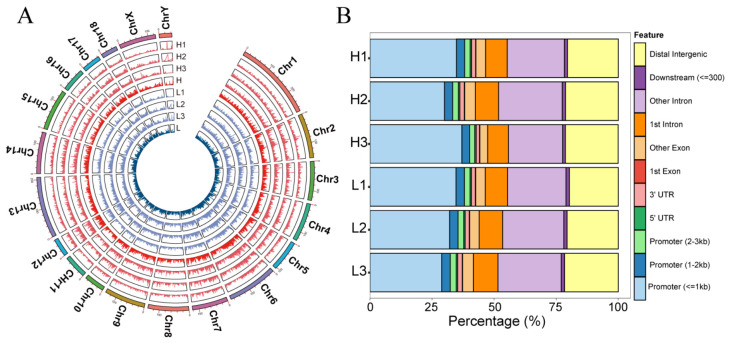
Identification and characterization of open chromatin peaks. (**A**) Genomic distribution of open chromatin peaks across porcine chromosomes. (**B**) Distribution of open chromatin peaks across genomic features.

A total of 82,488 and 85,682 ACRs were identified in the high- and low-IMF groups, respectively, of which 60,291 were shared between the two groups (Figure S2). Genome-wide analysis revealed that ACRs were distributed across nearly all porcine chromosomes, although relatively fewer peaks were detected on chromosomes X and Y (Figure 2A). Genomic annotation further showed that ACRs were predominantly located within intronic regions (mean 33.14%) and promoter regions (mean 32.91%), followed by intergenic regions (mean 20.82%) (Figure 2B). These results indicate that chromatin accessibility in porcine longissimus dorsi muscle is primarily concentrated in genomic regions associated with gene regulation and transcriptional activity.

### 3.4. Identification and Characterization of DACRs

**Figure 3:**
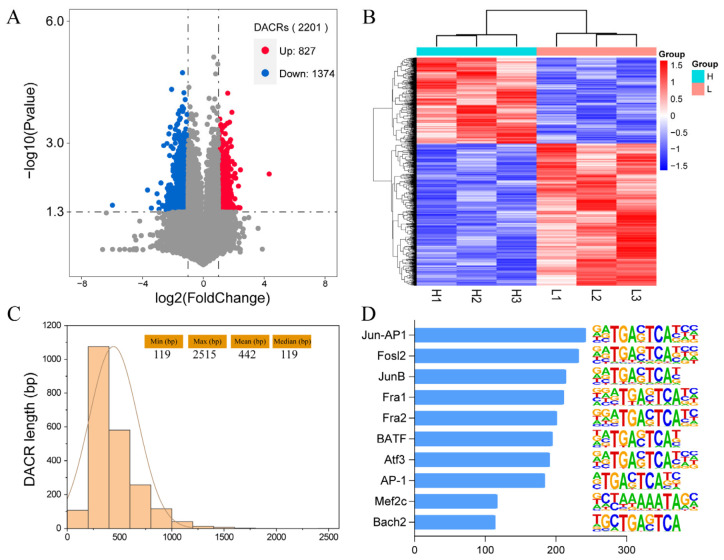
Identification and characterization of differentially accessible chromatin regions (DACRs) in the longissimus dorsi muscle of pigs with high and low IMF content. (**A**) Volcano plot of DACRs. (**B**) Hierarchical clustering heatmap of DACR accessibility profiles. (**C**) Length distribution of DACRs. (**D**) Top 10 significantly enriched transcription factor-binding motifs identified in DACRs.

To identify DACRs associated with IMF variation, differential accessibility analysis was performed on ACRs detected in the longissimus dorsi muscle of high- and low-IMF pigs using thresholds of *p* < 0.05 and |log_2_(fold change)| ≥ 1. A total of 2201 DACRs were identified, including 827 regions with increased accessibility and 1374 regions with decreased accessibility in the high-IMF group compared with the low-IMF group (Figure 3A). Hierarchical clustering analysis revealed that biological replicates from the same group clustered together, indicating highly consistent chromatin accessibility patterns within each group and clear separation between the two groups (Figure 3B). The lengths of the identified DACRs ranged from 119 to 2515 bp, with a mean length of 442 bp and a median length of 119 bp (Figure 3C). To further explore the regulatory factors associated with differential chromatin accessibility, motif enrichment analysis was performed on the DACRs. In total, 922 significantly enriched transcription factor-binding motifs were identified (*p* < 0.01). Among these, Jun-AP1, Fosl2, and JunB were the three most significantly enriched transcription factors (Figure 3D), suggesting their potential involvement in the regulation of IMF deposition.

### 3.5. RNA-Seq Data Quality Assessment and Mapping Statistics

To investigate transcriptional differences associated with IMF variation, RNA-seq was performed on longissimus dorsi muscle samples from Hezuo pigs with divergent IMF phenotypes. A total of 257,772,324 raw reads were generated from six libraries, of which 247,749,018 high-quality clean reads were retained after quality filtering. The sequencing data exhibited excellent quality, with mean Q20 and Q30 values of 98.64% and 96.10%, respectively, and an average GC content of 53.61%. Clean reads were subsequently aligned to the porcine reference genome, resulting in an average mapping rate of 92.80%, with individual samples ranging from 92.11% to 93.55% (Supplementary Table S3).

**Figure 4:**
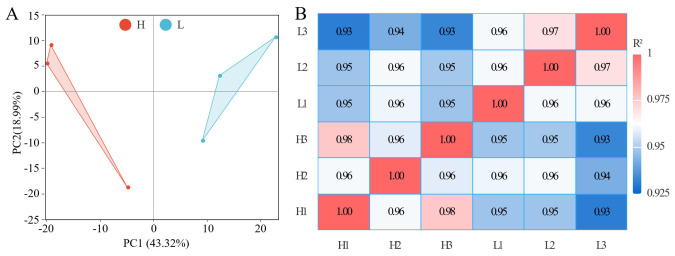
Overview of RNA-seq data. (**A**) Principal component analysis (PCA) of all samples. (**B**) Pearson correlation heatmap among samples.

To assess sample relationships and data reproducibility, principal component analysis (PCA) and Pearson correlation analysis were performed. The PCA clearly separated the high- and low-IMF groups along the first principal component (PC1), which explained 43.32% of the total variance, indicating distinct transcriptional profiles between the two groups (Figure 4A). Furthermore, samples within each group clustered closely together, demonstrating strong biological consistency. Pearson correlation coefficients ranged from 0.93 to 0.98 among all samples (Figure 4B), further supporting the high quality and reproducibility of the RNA-seq dataset.

### 3.6. Identification and Characterization of DEGs

**Figure 5:**
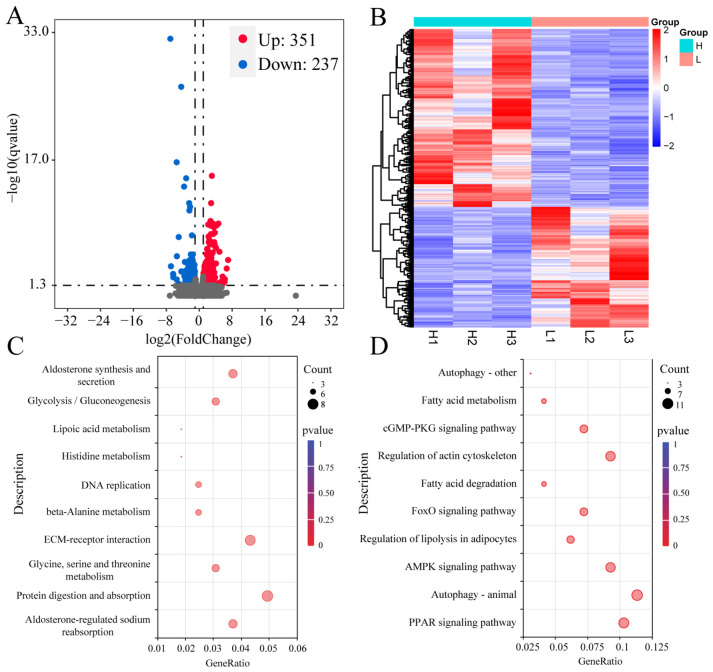
Identification and functional enrichment analysis of differentially expressed genes (DEGs). (**A**) Volcano plot of DEGs identified between the high- and low-IMF groups. (**B**) Hierarchical clustering heatmap of DEGs. (**C**,**D**) KEGG pathway enrichment analysis of significantly upregulated (**C**) and downregulated DEGs (**D**).

To identify genes associated with IMF variation in Hezuo pigs, differential expression analysis was performed on RNA-seq data from longissimus dorsi muscle samples using thresholds of q < 0.05 and |log_2_(fold change)| ≥ 1. A total of 588 DEGs were identified between the high- and low-IMF groups, including 351 upregulated and 237 downregulated genes in the high-IMF group (Figure 5A, and Supplementary Table S4). Hierarchical clustering analysis demonstrated that biological replicates from the same group clustered together, indicating highly consistent gene expression patterns within groups and clear transcriptional differences between the two phenotypes (Figure 5B).

To validate the RNA-seq results, six DEGs were randomly selected for qRT-PCR analysis. The expression patterns obtained by qRT-PCR were consistent with those identified by RNA-seq, confirming the reliability of the transcriptomic data (Figure S3). To further explore the biological functions of the identified DEGs, KEGG pathway enrichment analysis was performed. Upregulated genes were mainly enriched in pathways related to histidine metabolism, beta-alanine metabolism, and lipoic acid metabolism (Figure 5C). In contrast, downregulated genes were significantly enriched in pathways associated with fatty acid metabolism, fatty acid degradation, regulation of lipolysis in adipocytes, the AMPK signaling pathway, and the PPAR signaling pathway (Figure 5D). These findings suggest that genes involved in lipid metabolism and energy homeostasis may contribute to differences in IMF deposition between the two groups.

### 3.7. Integrated Analysis of ATAC-Seq and RNA-Seq Data

**Figure 6:**
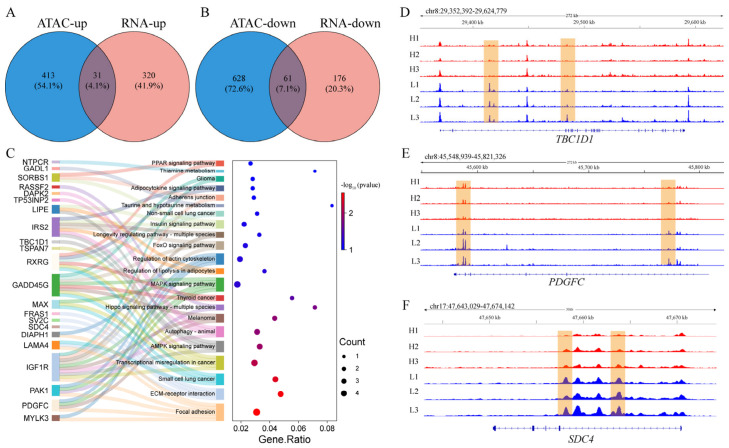
Integrated analysis of chromatin accessibility and gene expression in the high- and low-IMF groups. (**A**) Overlap between genes with significantly increased expression and genes associated with increased chromatin accessibility. (**B**) Overlap between genes with significantly decreased expression and genes associated with decreased chromatin accessibility. (**C**) KEGG pathway enrichment analysis of overlapping genes identified from the integrated ATAC-seq and RNA-seq analyses. (**D**–**F**) Genome browser tracks showing differentially accessible chromatin regions at the *TBC1D1*, *PDGFC*, and *SDC4* loci in the high- and low-IMF groups.

To investigate the relationship between chromatin accessibility and gene expression associated with IMF deposition, ATAC-seq and RNA-seq datasets were integrated. A total of 31 genes exhibited both increased chromatin accessibility and upregulated expression, whereas 61 genes showed concurrent decreases in chromatin accessibility and transcript abundance (Figure 6A,B). KEGG pathway enrichment analysis revealed that these co-regulated genes were primarily enriched in the ECM–receptor interaction, focal adhesion, and AMPK signaling pathways (Figure 6C). Several candidate genes associated with these pathways were identified, including *IGF1R*, *IRS2*, *MYLK3*, *PDGFC*, *PAK1*, *LIPE*, *LAMA4*, *DIAPH1*, *SDC4*, *SV2C*, *FRAS1*, *MAX*, *GADD45G* and *RXRG*.

To further examine chromatin accessibility at representative candidate loci, accessibility profiles were visualized using IGV. The ACRs associated with *TBC1D1* and *PDGFC* exhibited markedly stronger accessibility signals in the high-IMF group than in the low-IMF group (Figure 6D,E). Similarly, *SDC4* displayed distinct accessibility patterns between the two groups, with increased chromatin accessibility observed in high-IMF individuals (Figure 6F). These findings suggest that alterations in chromatin accessibility may contribute to the transcriptional regulation of genes associated with IMF deposition in Hezuo pigs.

## 4. Discussion

As an economically important trait, IMF content has a substantial influence on meat quality and sensory attributes in livestock, including flavor, tenderness, and juiciness [6,19,20]. Consequently, improving IMF content has become a major objective in modern livestock breeding programs. Although numerous candidate genes associated with IMF deposition have been identified, previous studies have primarily focused on transcriptomic analyses [21,22,23] and comparisons among different pig breeds [14]. In contrast, relatively little attention has been given to the regulatory mechanisms underlying IMF deposition from the perspective of chromatin accessibility, particularly in Hezuo pigs. Given that IMF deposition is a complex quantitative trait regulated by multiple genes and regulatory networks [24], integrated multi-omics approaches provide a powerful strategy for identifying key regulatory factors and elucidating the molecular mechanisms governing IMF accumulation.

Advances in high-throughput sequencing technologies have enabled the widespread application of ATAC-seq and RNA-seq for identifying accessible chromatin regions and DEGs associated with complex traits in livestock [25,26,27]. However, their combined application to investigate the regulatory mechanisms underlying IMF deposition in Hezuo pigs remains limited. In the present study, we integrated chromatin accessibility and transcriptomic profiling to characterize the molecular landscape associated with IMF variation in the longissimus dorsi muscle of Hezuo pigs with divergent IMF phenotypes.

Genome-wide analysis revealed that accessible chromatin regions were strongly enriched around TSSs and were predominantly located within intronic, promoter, and intergenic regions, consistent with previous reports [28]. Comparative ATAC-seq analysis identified 2201 DACRs, and motif enrichment analysis revealed significant enrichment of transcription factors associated with adipogenesis, including Jun-AP1, Fosl2, Fra1, Fra2, AP-1, and Mef2c. Specifically, Fra2 has been reported to regulate adipocyte dynamics through repression of PPARγ2, thereby influencing fat accumulation [29]. Similar transcription factors have also been identified in previous studies of IMF deposition in pigs [30,31].

RNA-seq analysis identified 588 DEGs, which were mainly enriched in pathways related to fatty acid metabolism, lipoic acid metabolism, and PPAR signaling. Previous studies have demonstrated that the PPAR signaling pathway plays a central role in lipid metabolism and pork quality regulation [32,33]. Integration of the ATAC-seq and RNA-seq datasets further identified 92 co-regulated genes enriched in focal adhesion and AMPK signaling pathways. Several candidate genes, including *IGF1R*, *IRS2*, *MYLK3*, *PDGFC*, *PAK1*, *LIPE*, *LAMA4*, *DIAPH1*, *SDC4*, *SV2C*, *FRAS1*, *MAX*, *GADD45G* and *RXRG*, were associated with these pathways. Among them, *IGF1R* has been shown to influence lipid accumulation and adipose tissue development [34,35], whereas IRS2 plays a critical role in growth, glucose homeostasis, and lipid metabolism through insulin and insulin-like growth factor signaling [36,37]. In addition, *GADD45G*, *LIPE*, and *RXRG* have previously been implicated in adipogenesis and fat deposition [15,38,39,40,41]. These findings suggest that coordinated changes in chromatin accessibility and gene expression contribute to IMF deposition in Hezuo pigs and highlight several promising candidate genes for future functional studies.

Collectively, the candidate genes identified in this study represent promising targets for understanding the molecular regulation of IMF deposition in pigs. These findings not only deepen our understanding of the genetic and epigenetic mechanisms underlying IMF accumulation but also provide valuable molecular resources for the development of marker-assisted breeding strategies aimed at improving meat quality traits. Nevertheless, several limitations of this study should be acknowledged. First, the relatively small sample size used for the omics analyses may have limited the statistical power of the study. Although qRT-PCR validation supported the reliability of the transcriptomic results, larger-scale studies are needed to further validate these findings and improve their generalizability. In addition, the regulatory functions of the identified candidate genes remain to be experimentally confirmed. Therefore, future investigations at the cellular and protein levels will be necessary to elucidate the precise roles of these genes in the regulation of IMF deposition.

## 5. Conclusions

In conclusion, this study integrated ATAC-seq and RNA-seq analyses to characterize genome-wide chromatin accessibility and gene expression patterns associated with IMF deposition in Hezuo pigs. A set of candidate genes potentially involved in lipid metabolism and fat deposition, including *IGF1R*, *IRS2*, *PAK1*, *LIPE*, *LAMA4*, *DIAPH1*, *SDC4*, *SV2C*, *GADD45G* and *RXRG*, was identified through integrated multi-omics analysis. These findings provide new insights into the molecular mechanisms underlying IMF deposition in indigenous pig breeds and offer valuable genetic resources for future studies and molecular breeding strategies aimed at improving pork quality.

## Figures and Tables

**Table 1:** Phenotypic characteristics of Hezuo pigs with high and low IMF content.

Traits	High-IMF	Low-IMF	*p*-Value
IMF (%)	3.64 ± 0.18	2.03 ± 0.11	0.0004
Body weight (kg)	15.53 ± 2.05	13.83 ± 1.35	0.38

Data are presented as mean ± standard deviation (SD). IMF, intramuscular fat.

**Table 2:** Summary statistics of ATAC-seq data from the longissimus dorsi muscle of Hezuo pigs.

Sample	Raw Reads	Clean Reads	Q20 (%)	Q30 (%)	Mapped Rate (%)	Uniquely Mapped Rate (%)
H1	92,201,589	90,913,431	98.25%	94.90%	95.00%	90.32%
H2	103,839,461	102,113,553	97.92%	93.94%	94.92%	91.58%
H3	59,471,348	59,079,876	99.07%	97.33%	95.80%	92.05%
L1	92,866,465	91,801,042	98.01%	94.10%	95.05%	90.56%
L2	87,567,781	86,264,231	97.95%	93.96%	93.19%	91.13%
L3	100,204,781	98,926,637	98.18%	94.67%	93.43%	89.99%

H, high-IMF group; L, low-IMF group. Q20 and Q30 represent the percentages of bases with Phred quality scores ≥20 and ≥30, respectively.

## Data Availability

All raw data generated in this study can be obtained from the corresponding author upon reasonable request.

## Supplementary Materials

The following supporting information can be downloaded at: https://www.mdpi.com/article/10.3390/ani16142172/s1, Figure S1: Distribution of insert sizes in six individuals; Figure S2: Venn diagram illustrates peak overlap between H and L groups; Figure S3: Validation of RNA-seq data by RT-qPCR. Table S1: qPCR primers for validating DEGs in the longissimus dorsi muscle of low- and high-IMF Hezuo pigs; Table S2: Phenotypic characteristics of Hezuo pigs with high and low IMF content. Table S3: Summary statistics of the RNA-Seq data; Table S4: Differentially expressed genes in the longissimus dorsi muscle between low- and high-IMF Hezuo pigs.

## Abbreviations

The following abbreviations are used in this manuscript:

ATAC-seq	assay for transposase-accessible chromatin using sequencing
DACR	differential accessible chromatin region
DEG	differentially expressed gene
IMF	intramuscular fat
PCA	principal component analysis
RNA-seq	RNA sequencing
TSS	transcription start site
