# Supplementary material for: Integrated ATAC-Seq and RNA-Seq Reveal Candidate Regulatory Genes and Chromatin Accessibility Associated with Intramuscular Fat Deposition: An Animal Trial in Hezuo Pigs
Source: Animals (Basel). 2026 Jul 13;16(14):2172. doi: 10.3390/ani16142172 (PMC13403781; doi:10.3390/ani16142172)
Supplement: Supplementary file 1 [file animals-16-02172-s001.zip › animals-4381099-supplementary.pdf]

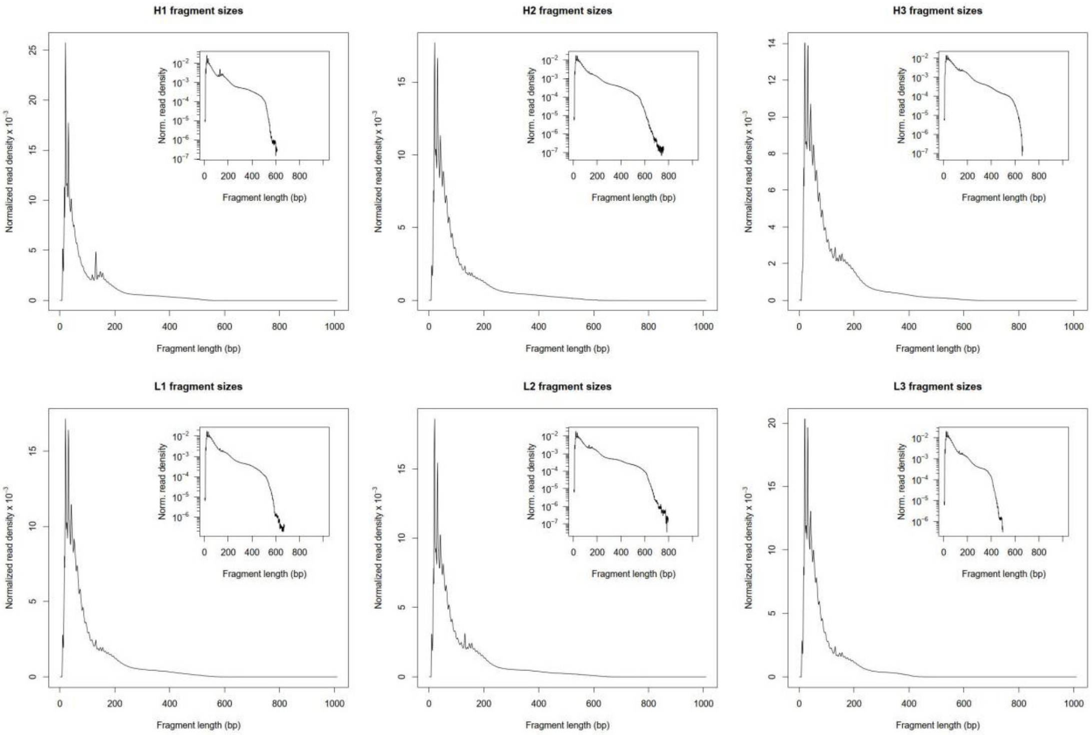

Figure S1 Distribution of insert sizes in six individuals

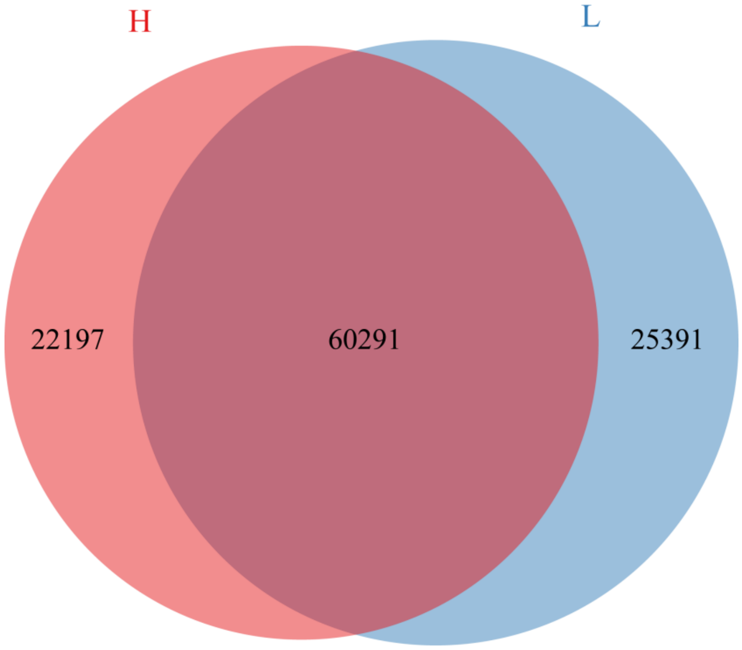

Figure S2 Venn diagram illustrates peak overlap between H and L groups

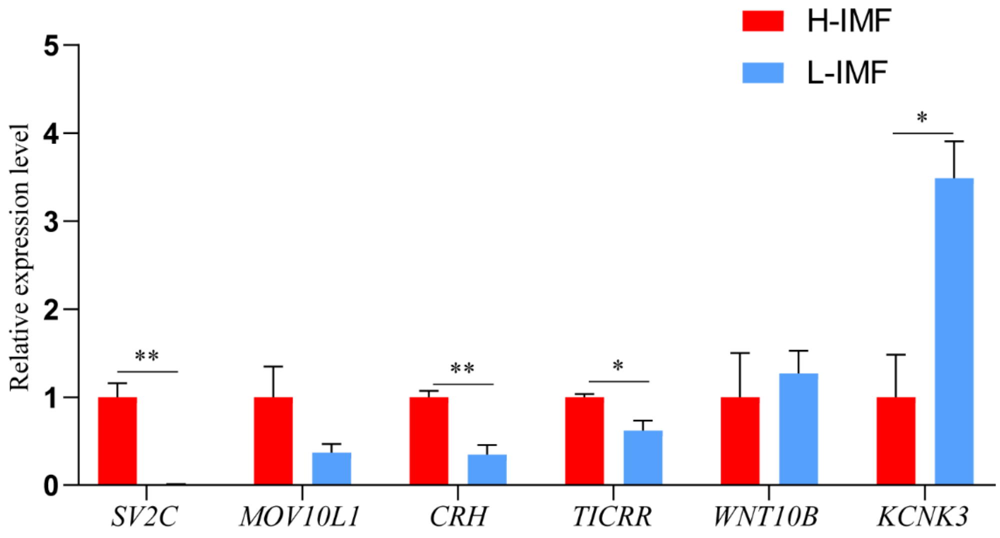

Figure S3 Validation of RNA-seq data by RT-qPCR. Relative expression levels measured by the y-axis RT-qPCR. Mean ± standard deviation, * represents $p < 0.05$; ** represents $p < 0.01$.

Table S1 qPCR primers for validating DEGs in the longissimus dorsi muscle of low- and high-IMF Hezuo pigs

**Gene name**	**Primer**	**Length (bp)**
SV2C-F1	CAAAGATTCCGTTTTCAAGTCCT	191
SV2C-R1	CATCATCAAAGGTAATTGGGCAT	
MOV10L1-F1	GCTACGTCGTCGAGATTCGT	142
MOV10L1-R1	TGCTCAAGGGCAAAGTGACA	
CRH-F1	AGGCTCTGGTGTGGAGAAAC	`193
CRH-R1	GCATTTTAGGGGCGCTTCTG	
TICRR-F1	GAGGTGTTTGCTTCAGGTGC	122
TICRR-R1	GAGGAGGGCATCTTCGTCTG	
WNT10B-F1	TATTCCGGAGCCCTCCTTGT	169
WNT10B-R1	ATTGCTTAGGGCCCGACTG	
KCNK3-F1	TCGGCTTCTTCTCGTGCATT	189
KCNK3-R1	AGCTAAAGGCCACGTACTGC	
GAPDH-F1	AGTATGATTCCACCCACGGC	139
GAPDH-R1	TACGTAGCACCAGCATCACC	

Table S2 Phenotypic characteristics of Hezuo pigs with high and low IMF content

Sample	Sex	Body weight	IMF
H1	male	14.5	3.44
H2	male	13.7	3.62
H3	male	18.4	3.87
L1	male	12.2	2.14
L2	male	13.8	2.07
L3	male	15.5	1.88

Table S3 Summary statistics of the RNA-Seq data

sample	raw_reads	clean_reads	Q20 (%)	Q30 (%)	GC content (%)	Mapped rate (%)
H1	40806972	38930988	98.62	96.01	53.64	92.69
H2	42250372	40368926	98.61	95.99	53.34	92.83
H3	41524228	40063880	98.61	95.95	54.9	93.19
L1	44874250	43252284	98.72	96.30	53.98	93.55
L2	45973490	44319390	98.70	96.32	53.08	92.42
L3	42343012	40813550	98.6	96.01	52.70	92.11

Table S4 Differentially expressed genes in the longissimus dorsi muscle between low- and high-IMF Hezuo pigs

Gene name	$log_2^{FoldChange}$	q_value
SV2C	7.025533053	3.36E-05
MOV10L1	6.679023066	0.000392749
CILP2	6.235338182	0.008626005
LOC110260203	6.165660515	0.018095412
LOC110260668	5.825369628	0.009276197
LOC110257444	5.685008376	0.004019344
HSD11B2	5.549234437	0.035453494
LOC100513319	5.419317836	0.043566613
CRH	5.282627033	0.018609314
TICRR	5.12178813	0.027069076
LOC100524923	4.918607957	0.047763611
PKHD1L1	4.705207014	0.027289455
LOC110257281	4.666129899	9.11E-10
LOC102160916	4.565033994	0.035808764
NR5A2	4.486773143	0.029032463
HOXD10	4.478325303	0.042765602
FAM83D	4.280504703	0.042873502
KCP	4.175140998	1.61E-09
C1QTNF6	4.087850725	9.19E-05
SFRP2	4.083628735	1.03E-06
MDFI	3.845278901	0.005170088
SUSD1	3.799782934	1.21E-08
BUB1B	3.792354965	0.027530626
POLE	3.771209	0.006010819
CPXM1	3.735715224	4.05E-07
LOC100522421	3.734389774	5.48E-07
LOC110261095	3.673013588	0.001263498
PIMREG	3.667513031	0.035559223
LOC396781	3.648221995	1.93E-07
CRISPLD1	3.628575588	0.001172554
ADAMTSL1	3.556174088	2.60E-09
UBE2C	3.537029981	0.01317512
SCD	3.509716894	0.003615355
APLNR	3.381082883	1.87E-08
ADAMTS16	3.373522525	0.000354413
LOC110261088	3.338409944	0.000941744
FBLN7	3.333371427	0.002373952
RPRM	3.321494593	9.11E-10
COL6A6	3.290971187	3.97E-06
MCM10	3.290829897	0.049512554
KIF20A	3.225332167	0.002271108

Gene name	$log_2^{FoldChange}$	q_value
TCF19	3.220729759	0.02228418
ADAM12	3.217924583	0.027091939
TOP2A	3.161845695	0.000449643
LOC100523747	3.124427401	0.000127928
KERA	3.085320594	0.032171613
KY	3.071043936	9.37E-16
ERFE	3.053803935	0.017409516
ADAMTS17	3.033554387	0.001075639
E2F1	3.02919447	0.003068655
FGF18	3.01509041	0.024587564
IGFBP5	2.990594747	5.44E-05
CCRL2	2.9895452	0.01482043
AQP3	2.987358772	7.24E-06
PBX3	2.982662851	9.82E-10
OPCML	2.935895686	1.93E-05
ROBO1	2.917992676	0.007753913
SAMD11	2.889513586	2.50E-12
PLPPR5	2.87142054	0.01166428
MYBL2	2.870782507	0.003711007
ARG2	2.867714977	1.87E-08
UHRF1	2.862561765	0.009431952
ABCD2	2.846490619	0.004585034
CRYM	2.843376781	0.016022461
PLEKHD1	2.823000821	0.017660422
SHISA2	2.790045745	1.60E-09
LOC106507442	2.783530986	0.000118373
EPHB1	2.782856661	0.004144754
LRRC17	2.779421674	0.003975044
RCOR2	2.757244361	4.36E-06
MIR6782	2.747134368	0.023015406
LOC106505661	2.745696235	2.87E-05
RXRG	2.734063011	4.34E-10
MKI67	2.713463575	0.000538082
FAM46C	2.677810647	0.000186112
GADL1	2.633342175	3.76E-07
KCNIP3	2.629894183	0.047325911
RGS14	2.621104031	3.36E-05
RN5-8S	2.600493324	0.009786452
RASSF2	2.593390359	0.003068655
FMN1	2.591308452	0.006816918
COL1A2	2.587628485	0.000202583
SLC16A14	2.575952873	0.004257071
MCM2	2.562308074	0.031466428

Gene name	$log_2^{FoldChange}$	q_value
PSPH	2.50533493	1.77E-05
ARL11	2.49336727	0.015346859
CFAP54	2.482237603	0.014640906
FRRS1	2.467006099	1.04E-07
KCNK13	2.451677636	0.027920041
LOC110256424	2.430869115	0.001061127
CCDC80	2.427414972	3.60E-06
CREB3L1	2.423563275	0.003431205
LOC110259958	2.401421831	0.036795808
PALD1	2.39001894	0.00605999
GRID2IP	2.374848841	0.000170732
ART4	2.365139711	0.009148081
MCM3	2.364600195	0.00601789
MATN4	2.350905159	0.030216388
LOC106508719	2.346000816	1.85E-05
RELT	2.337517758	0.00061901
SPOCK2	2.321583052	1.93E-07
COL3A1	2.312313076	0.000529045
SLC5A4	2.307275284	0.000566063
MMP23B	2.303247171	0.021662885
KCNS3	2.293936316	0.000186112
SNTB1	2.281480577	3.78E-08
MYOD1	2.28040077	6.46E-05
CIDEC	2.280023901	0.02973432
HOXC10	2.243327376	0.002776633
CD109	2.237996351	0.00221168
RNF165	2.22204898	0.027530626
FBXL22	2.211163818	0.005360005
MAPK4	2.195664634	0.008704042
SLC7A5	2.194380328	1.22E-09
HNMT	2.178129994	1.90E-05
BMPER	2.169216205	0.001663393
STAC	2.13709809	0.000810246
ST8SIA2	2.134166605	0.007718711
DBP	2.124131001	0.000186112
CDO1	2.120198	0.001170016
TCHP	2.11781905	0.000366693
EMILIN3	2.110248561	0.000942827
COL21A1	2.107294889	0.000874354
ABCB9	2.086578948	0.008219178
LOC110260469	2.074794097	0.044373066
LAMB3	2.07193782	0.036803706
LOC110257935	2.071869969	0.007848648

Gene name	$log_2^{FoldChange}$	q_value
TACC3	2.067479876	0.028617932
CKAP2	2.057943721	0.015449167
APOE	2.056376377	0.00086495
RET	2.055972788	0.003448581
LOC106506850	2.042943002	0.002508458
FSCN1	2.038237143	0.00075834
ADAMTS4	2.030327678	0.048179787
ANGPT1	2.006664315	0.00044018
LXN	2.001637514	0.01071554
RNF212	1.998830604	0.009117291
DEPTOR	1.993899597	4.36E-06
LRRN4CL	1.993291573	0.000165679
SRPX	1.989246008	0.034464236
IGF1	1.984303527	0.000529045
DHCR24	1.970802332	0.000854184
LOC110255581	1.970476279	0.001803771
MEGF10	1.967632145	0.004689134
LOC102165485	1.966788977	1.54E-06
AMT	1.942671592	0.018609314
SEPT6	1.942601183	0.000203732
CACNB3	1.940739577	0.017688807
FMOD	1.927092267	0.013671586
ATP1B2	1.926360732	0.000186112
CXCL14	1.917603057	0.003008388
IQCK	1.917249828	1.35E-06
TET1	1.901390345	0.000529045
PODXL2	1.880790849	5.77E-05
PLK2	1.87328196	0.004801024
SOX8	1.856268198	0.005673149
PENK	1.853825368	0.044734939
MLEC	1.83357946	5.92E-05
NTRK2	1.832097355	0.005398768
PPP1R3D	1.828031884	0.004019344
SUOX	1.827708288	0.001861077
TENM4	1.82664155	0.001803771
FAM78A	1.826131667	0.000813248
SLC37A4	1.821149743	0.000717877
LOC110261482	1.820200782	7.24E-06
PSAT1	1.801498724	0.033344475
LOC100737961	1.777368577	0.00440468
TIMP4	1.775272366	0.031466428
LINGO1	1.766139633	0.00023915
SPATS2	1.756849111	0.000115238

Gene name	$log_2^{FoldChange}$	q_value
TMEM51	1.754823685	0.021481199
PAK1	1.745454258	0.018107816
CDC42BPG	1.739367831	0.020529878
ELOVL6	1.738933721	0.014594131
SLC7A8	1.731720452	0.016091039
CYP3A29	1.730225874	0.044871492
LSMEM1	1.728869837	0.001066381
PRKAG3	1.726074216	0.000113569
LOC106504851	1.725844605	0.002512675
ST3GAL5	1.718688309	5.37E-05
LUM	1.704346641	0.023585207
LOC106504670	1.701106208	0.047351437
FST	1.69959627	0.026839722
SH3RF2	1.70	0.000170732
CARNS1	1.687045597	0.001903318
NQO1	1.678475324	0.000474255
COL5A2	1.675959603	0.000727862
PHOSPHO1	1.674553666	0.003431205
LHX6	1.669520752	0.044725908
VASH1	1.662884703	0.033344475
GINS2	1.662792505	0.007526672
FAM212B	1.659917528	0.000545782
ANGPTL1	1.658727108	0.004909728
SYT12	1.648828698	0.01071554
MARCKS	1.642006474	0.012533674
ZNF704	1.632426173	0.010273823
POLH	1.631802882	0.001977054
EGF	1.627983216	0.031428962
PRC1	1.622014011	0.03923288
PTGFRN	1.620813564	0.011973811
ANKRD28	1.615379321	0.000727862
PLXDC1	1.614643366	0.021677172
ABI3BP	1.614468371	0.000381009
FAM117A	1.614467616	0.002039183
LOC102162205	1.609173489	0.00183093
HECTD2	1.608679799	0.027356676
ARSB	1.604301573	0.008765123
GPD1	1.602212663	0.001181939
CNTFR	1.601281139	0.001026804
LY6E	1.593850142	0.001466656
FAM71E1	1.589886801	0.026375878
NOX5	1.587816785	0.031466428
SULT1A3	1.585871826	0.002886938

Gene name	$log_2^{FoldChange}$	q_value
LOC110258600	1.582147374	0.02218278
CCDC28B	1.576411938	0.022294459
WNT4	1.56264486	0.001263274
VOPP1	1.55974614	0.001458383
SERPINF1	1.55546493	0.00435423
ANKRD34A	1.554321019	0.016994539
OLFML2B	1.548654025	0.018120497
RHOBTB2	1.547018195	0.003301793
GRASP	1.540901219	0.001531524
LSS	1.537804883	0.002463853
AGTPBP1	1.531025074	0.006010819
HLF	1.521876068	0.024501949
KLHL25	1.513565388	0.030216388
RCN1	1.507465538	0.027882602
LOC102168016	1.506389182	0.029675967
SEMA4D	1.501486133	0.000122411
MMD	1.494583058	0.010200825
DMPK	1.494354413	0.00435423
SEC24D	1.472730785	0.000127928
PI15	1.462319276	0.04891059
FRAS1	1.461714822	0.004346106
RHOBTB1	1.461309035	0.012424382
PGM2L1	1.457345852	0.001064126
EYA1	1.455229535	0.00240895
RRBP1	1.447652231	0.002448787
LRRN1	1.4465208	0.002107132
CUNH14orf80	1.440739208	0.005359563
PDK3	1.436361754	0.049130671
SLC16A5	1.435098368	0.012124808
SMYD1	1.433836401	0.004689134
SSC5D	1.432088644	0.008626005
STBD1	1.425724525	0.001866769
RAB31	1.415226148	0.00907442
COL15A1	1.414026984	0.006356516
EEF2K	1.406017524	0.010726509
MAP2K6	1.402473334	0.002712953
HLCS	1.399541798	0.001918614
OGN	1.394064486	0.022156405
PYCR1	1.388091405	0.0086455
STAT5A	1.378266043	0.00045176
MSRB1	1.378065023	0.036795808
CAMKK1	1.374519664	0.004585034
PLXND1	1.368727179	0.035153125

Gene name	$log_2^{FoldChange}$	q_value
GPRC5B	1.363513838	0.001458383
STMN1	1.362699242	0.023463273
NAV2	1.36121199	0.048179787
ATP1A1	1.356392352	0.005055201
SEL1L3	1.354572014	0.004172506
RNFT1	1.349051629	0.000157037
OLFML2A	1.346830575	0.009034366
MVB12B	1.343840204	0.001567238
PGM3	1.341592242	0.005290764
TTYH2	1.339007429	0.007377396
NFE2L3	1.333625033	0.013042975
PER3	1.327135757	0.033701163
DZIP1	1.325818002	0.010093312
TDRD7	1.325768259	0.002073952
ZNRF3	1.31242054	0.004588688
EHD1	1.312368716	0.001147171
LOC396711	1.310316056	0.026839722
ANO1	1.309889288	0.004368331
FAM198B	1.306291906	0.01418799
EML1	1.301904377	0.008063243
KCTD15	1.301103417	0.001861077
MRC2	1.300524717	0.015433705
FNBP1	1.294356585	0.012124808
CDKN2C	1.292328218	0.005599437
MTUS1	1.291921555	0.015449167
CRYBG3	1.290254046	0.037038596
ASB1	1.287441102	0.00023915
EPDR1	1.283991615	0.005853382
AMPD1	1.280671979	0.00023915
AGL	1.278805542	0.034464236
IVD	1.277114789	0.005843065
FRMD4B	1.265737967	0.047351437
LBX1	1.252459912	0.015449167
HOMER1	1.247976443	0.033344475
SLC16A3	1.247842633	0.000727862
CAPN6	1.245164546	0.049130671
SGMS2	1.242172115	0.015887154
LDHA	1.241785811	0.009034366
DAGLA	1.24	0.017088197
FAM184B	1.238052147	0.036524233
MKL1	1.236558767	0.035808764
NUDT4	1.232850532	0.017179429
PDGFRL	1.219669925	0.032899207

Gene name	$log_2^{FoldChange}$	q_value
MYOC	1.219219148	0.026839722
DENND1B	1.218933736	0.048179787
RGS12	1.217859063	0.025607649
PRKRA	1.214807796	0.037757002
NMNAT3	1.21159608	0.019795985
LAMA4	1.206315211	0.026839722
NTPCR	1.206167337	0.005252666
P2RY2	1.203302833	0.032171613
EXTL1	1.198540496	0.006808383
RHBDL1	1.188745545	0.010765499
PTPRS	1.182292589	0.002448787
CPE	1.180982717	0.034960733
MUM1L1	1.179244959	0.026272727
GALNT17	1.177186443	0.044252301
EPB41	1.176090434	0.00563643
TMEM233	1.173491077	0.013919289
TRIM7	1.15	0.0104447
CASQ1	1.149063302	0.010093312
PGM1	1.143932048	0.017131708
TMEM246	1.143191735	0.012917886
TRO	1.136036893	0.049360531
TNFRSF19	1.134436813	0.035377627
RNASEH2B	1.13408012	0.015058428
PCDH12	1.126735444	0.044308891
FAM129A	1.126302081	0.041611108
GPI	1.124291943	0.013416814
NEDD4L	1.123752932	0.015693746
PKM	1.12024503	0.019498149
PLXNA1	1.119251052	0.011326669
ALDH1L2	1.117258692	0.029824505
PEBP4	1.116887705	0.003819033
FREM2	1.115856605	0.046699304
KPNA5	1.110008858	0.027920041
CSK	1.109624667	0.007214208
SERPINH1	1.103862544	0.026582761
PIP4K2A	1.091578086	0.030216388
ZNF512B	1.090252036	0.003149963
RGMA	1.087022896	0.019523924
IRS1	1.080590464	0.022156405
ANK1	1.079378781	0.011903609
MAMSTR	1.076020265	0.01418799
ADSSL1	1.065398679	0.029824505
EHHADH	1.062872006	0.008561553

Gene name	$log_2^{FoldChange}$	q_value
MAN2A1	1.057310952	0.048179787
MAFA	1.051278778	0.02779914
STARD10	1.049679346	0.007060374
MYBPC2	1.047393428	0.026560472
MYOG	1.036715584	0.040762433
TBC1D10B	1.032911279	0.001613079
PFKFB4	1.031000195	0.048179787
TSPAN7	1.025607878	0.031649558
UBE2E3	1.00929427	0.012020847
LETM1	-1.01	0.010726509
OXNAD1	-1.02	0.005986697
C7H6orf136	-1.03	0.017088197
TNFSF10	-1.03	0.038765271
POLR3GL	-1.03	0.041734953
ALPK3	-1.03	0.00907442
PPP1R12C	-1.04	0.032171613
CPT2	-1.04	0.012035659
FILIP1	-1.04	0.000884425
TRAF6	-1.04	0.048179787
MRPL47	-1.06	0.031466428
PDLIM3	-1.06	0.021481199
FDX1	-1.07	0.009987115
LOC100525935	-1.07	0.031466428
DPYSL2	-1.07	0.027644556
PRKD1	-1.07	0.031466428
ACOT9	-1.08	0.019795985
BFAR	-1.09	0.006843532
C4H8orf33	-1.09	0.008672944
FRG1	-1.10	0.046844158
SLC25A4	-1.10	0.00185917
KLHL41	-1.11	0.004801024
DUSP26	-1.12	0.00965559
LOC733637	-1.12	0.010200825
HTATIP2	-1.12	0.043323596
ACTN2	-1.13	0.009754521
MDH1	-1.13	0.000134945
MAX	-1.13	0.04992586
HSDL2	-1.14	0.010273823
ART1	-1.14	0.00374501
UTP3	-1.15	0.010726509
HIGD1A	-1.16	0.034459333
PDE7B	-1.16	0.027824867
PDGFC	-1.17	0.04353193

Gene name	$log_2^{FoldChange}$	q_value
CPT1B	-1.18	0.007349947
CHRNB1	-1.18	0.011786679
ETFDH	-1.18	0.005843065
PPP2CA	-1.19	0.016633846
LRPAP1	-1.19	0.011373556
HSPB3	-1.19	0.015957601
UBC	-1.20	0.02005582
ATOH8	-1.20	0.011727314
ESRRA	-1.21	0.000884425
WDR45	-1.21	0.003819033
MAP2K3	-1.21	0.001876137
MMP11	-1.22	0.014594131
BAG3	-1.22	0.013722497
LOC100152714	-1.22	0.033328476
ABCB6	-1.23	0.010726509
GPAT4	-1.23	0.007377396
GK	-1.23	0.048179787
IGF1R	-1.24	0.031906339
DES	-1.24	0.000362052
ADRB2	-1.25	0.029675967
TRPV2	-1.25	0.011373556
LDHB	-1.25	0.015310128
PNPLA2	-1.26	0.002515767
TBC1D1	-1.26	0.00240895
LOC100156657	-1.27	0.015058428
PLA2G16	-1.27	0.02050256
PRRX1	-1.29	0.000347256
EIF4E3	-1.29	0.003301793
WIPI1	-1.29	0.003821637
ART3	-1.31	0.028831697
ZNF275	-1.31	0.027920041
CHRNA10	-1.31	0.01923784
CDPF1	-1.31	0.004689134
PANK1	-1.31	0.029824505
MME	-1.33	0.010273823
BTNL9	-1.33	0.044084613
MTURN	-1.33	0.031466428
MIGA2	-1.33	0.001803771
ITIH4	-1.34	0.026592
LOC110256043	-1.34	0.017179421
ACADVL	-1.34	0.004689134
NFE2L1	-1.34	0.004643249
ACSL1	-1.35	0.01071554

Gene name	$log_2^{FoldChange}$	q_value
HOMER2	-1.35	0.027127839
TCAP	-1.37	0.008919335
ZNF697	-1.38	0.044734939
RNF115	-1.38	0.001808855
BMP1	-1.39	0.000253782
FAM167B	-1.39	0.028137276
IL13RA1	-1.39	0.020155121
PROSER2	-1.40	0.003819033
DIAPH1	-1.40	0.011719277
CEP135	-1.41	0.04871861
TWF2	-1.42	0.000474255
ADAMTSL4	-1.42	0.044084613
METRNL	-1.43	0.025607649
TBC1D8	-1.43	0.022623537
ARRDC2	-1.43	0.000703589
KCNQ4	-1.43	0.000517074
SLA-1	-1.44	0.009786452
FHL1	-1.44	0.000411108
ABHD18	-1.45	0.044084613
GLUL	-1.46	0.004643249
MARC2	-1.46	0.006096268
PLA2G7	-1.46	0.002776633
ENAH	-1.47	0.00474964
ITIH3	-1.48	0.046660758
PLIN2	-1.48	0.047351437
PPP1R27	-1.48	0.003766079
GADD45G	-1.49	0.000706702
PXDC1	-1.49	0.029626453
DAPK3	-1.50	0.035432663
IRS2	-1.50	0.027091939
PFN2	-1.51	0.00078069
RNF41	-1.51	0.001244568
UGP2	-1.52	0.000783087
LOC100736765	-1.52	0.029675967
TP53INP2	-1.54	0.004215659
TENM3	-1.54	0.001263498
ACTR3B	-1.54	5.44E-05
CASQ2	-1.55	6.81E-06
LOC106504574	-1.57	0.021783294
APOD	-1.58	0.007063728
HOOK2	-1.58	0.001197504
WFS1	-1.59	0.001861077
ZNF385D	-1.60	0.000979662

Gene name	$log_2^{FoldChange}$	q_value
TINAGL1	-1.62	3.33E-05
LOC100624650	-1.62	0.001808855
CRYAB	-1.62	0.00185917
FAS	-1.63	0.049735918
SLC27A4	-1.63	0.017179429
RGS4	-1.63	0.040542502
LOC110255568	-1.66	0.008348646
LOC102159510	-1.67	0.027069076
SDC4	-1.67	0.01933446
TECPR1	-1.69	0.044252301
PCGF6	-1.70	0.006308795
ABTB2	-1.71	0.009409167
PFKL	-1.72	0.001866769
RASGEF1B	-1.73	0.038188114
TTC9	-1.74	0.003590855
DNAJA4	-1.75	1.95E-05
HMOX2	-1.77	0.00146893
ATP2B3	-1.77	0.000355241
DAPK2	-1.77	2.73E-08
MLLT11	-1.78	2.06E-05
ABHD4	-1.80	1.94E-05
PRSS8	-1.80	0.00677718
SLC2A5	-1.80	0.027668289
FABP3	-1.80	0.002925339
ACSS1	-1.82	0.000590536
IGFBP3	-1.82	0.033360768
LOC110256117	-1.83	0.015467181
INSR	-1.85	0.000717877
NPR3	-1.86	0.003615355
LMCD1	-1.87	0.000230294
DDAH1	-1.87	0.002459348
PPM1M	-1.88	0.016022461
LOC110258338	-1.90	0.010200825
LIPE	-1.90	0.006928964
ZNF774	-1.92	0.010093312
FKBP5	-1.92	0.00156336
XIRP1	-1.92	0.001147171
LMOD2	-1.93	0.000428589
GABARAPL1	-1.93	0.001977054
SORBS1	-1.95	0.00478711
TPPP3	-1.96	0.031906339
MYH10	-1.96	8.59E-05
RAB30	-1.96	0.043853223

Gene name	$log_2^{FoldChange}$	q_value
IL6R	-2.00	0.003819033
C8H4orf46	-2.02	0.000314638
MYOM3	-2.02	0.00146893
HSPA2	-2.02	0.002463993
ADGRF2	-2.03	0.040542502
CNKSR2	-2.04	6.81E-06
SLC4A11	-2.05	0.007083292
DRP2	-2.06	0.015736252
LOC100525027	-2.07	0.019498149
BAALC	-2.10	0.044252301
NCMAP	-2.10	0.034798926
TMEM86A	-2.11	0.016298188
LOC110255237	-2.13	0.004643249
ADAMTSL5	-2.14	0.007063728
LOC102161634	-2.15	0.025663668
AGBL1	-2.18	0.001119592
LOC100523131	-2.19	0.001728588
SMIM3	-2.20	0.001147171
KLF11	-2.21	0.010333923
PLIN5	-2.22	7.15E-12
ADGRG6	-2.22	0.006999407
SMPDL3A	-2.23	0.003754096
CNTNAP5	-2.24	0.018192475
CYP4F55	-2.25	0.00115042
SYNJ2	-2.31	0.002776633
CCDC71L	-2.33	6.46E-05
HSPB7	-2.36	3.61E-05
FNTB	-2.38	2.07E-11
LOC100739101	-2.39	7.10E-05
ABHD2	-2.42	0.000118847
SMTNL1	-2.44	2.50E-12
MLYCD	-2.45	0.000772367
NRCAM	-2.45	0.017409516
ANXA8	-2.56	0.007544018
KLHL34	-2.63	0.000203732
LOC110257572	-2.63	0.000203732
SRXN1	-2.64	0.000432034
LOC102165817	-2.65	0.00677718
LOC100521322	-2.65	0.000299558
TNFRSF12A	-2.75	6.50E-05
LOC110260634	-2.75	0.00626642
C15H2orf88	-2.78	9.19E-05
CPLX1	-2.82	9.23E-05

Gene name	$log_2^{FoldChange}$	q_value
MMP25	-2.93	0.00435423
FAM83G	-2.99	0.026582761
LOC110255727	-3.01	0.002665384
CRTAC1	-3.02	0.00367421
LOC102167481	-3.05	0.041734953
RAB3B	-3.12	0.000727862
IRX3	-3.16	1.90E-15
LOC102168165	-3.19	0.010200825
STX3	-3.23	0.010726509
LOC110260631	-3.26	0.000741757
LOC100158115	-3.34	0.016024177
MIR208B	-3.38	0.023024246
LOC110256168	-3.38	0.007437313
S100A2	-3.47	0.044084613
MYLK3	-3.49	5.92E-05
LOC110257555	-3.61	0.003537855
CDK18	-3.68	2.10E-14
GALNT15	-4.34	0.000886876
LOC102168177	-4.36	0.000924235
CSRP3	-4.38	6.47E-27
LOC102163231	-4.87	0.034597831
ARMC12	-4.98	4.43E-08
LOC110257276	-5.25	0.047108302
WNT10B	-5.28	0.04547889
LOC106505879	-5.43	1.13E-05
KCNK3	-5.47	0.009290077
LOC100624137	-5.48	1.86E-17
LOC102159627	-6.30	0.004991855
LOC100512780	-6.34	0.001861077
LOC110260888	-6.87	0.000203732
PRSS36	-7.02	6.10E-33
